# Trends in Race/Ethnicity Among Applicants and Matriculants to US Surgical Specialties, 2010-2018

**DOI:** 10.1001/jamanetworkopen.2020.23509

**Published:** 2020-11-02

**Authors:** Edwin Nieblas-Bedolla, John R. Williams, Briana Christophers, Christopher Y. Kweon, Estell J. Williams, Nathalia Jimenez

**Affiliations:** 1 University of Washington School of Medicine, Seattle; 2 Department of Neurological Surgery, University of Washington, Seattle; 3 Weill Cornell Medicine–Rockefeller–Sloan Kettering Tri-Institutional MD-PhD Program, New York, New York; 4 Department of Orthopaedics and Sports Medicine, University of Washington, Seattle; 5 Department of Surgery, University of Washington, Seattle; 6 Department of Anesthesiology and Pain Medicine, University of Washington, Seattle

## Abstract

**Importance:**

Surgical programs across the US continue to promote and invest in initiatives aimed at improving racial/ethnic diversity, but whether this translates to changes in the percentage of applicants or matriculants from racial/ethnic minority groups remains unclear.

**Objective:**

To examine trends in the percentage of applicants and matriculants to US surgical specialties who identified as part of a racial/ethnic group underrepresented in medicine from the 2010-2011 to 2018-2019 academic years.

**Design, Setting, and Participants:**

This cross-sectional study examined trends in self-reported racial/ethnic identity among applicants and matriculants to US residency programs to evaluate demographic changes among surgical programs from 2010 to 2018. Data were obtained from the Association of American Medical Colleges.

**Results:**

The study population consisted of a total of 737 034 applicants and 265 365 matriculants to US residency programs, including 134 158 applicants and 41 347 matriculants to surgical programs. A total of 21 369 applicants (15.9%) and 5704 matriculants (13.8%) to surgical specialties identified as underrepresented in medicine. There was no statistically significant difference in the percentage of applicants underrepresented in medicine based on race/ethnicity for all surgical specialties combined in 2010 vs 2018 (15.3% [95% CI, 14.7%-15.9%] vs 17.5% [95% CI, 16.9%-18.1%]; *P* = .63). Thoracic surgery was the only surgical specialty in which there was a statistically significant change in the percentage of applicants (8.1% [95% CI, 4.9%-13.2%] vs 14.6% [95% CI, 10.2%-20.4%]; *P* = .02) or matriculants (0% [95% CI, 0%-19.4%] vs 10.0% [95% CI, 4.0%-23.1%]; *P* = .01) underrepresented in medicine based on race/ethnicity. Obstetrics and gynecology had the highest mean percentage of applicants (20.2%; 95% CI, 19.4%-20.8%) and matriculants (19.0%; 95% CI, 18.2%-19.8%) underrepresented in medicine among surgical specialties. Thoracic surgery had the lowest mean percentage of applicants (12.5%; 95% CI, 9.46%-15.4%) and otolaryngology the lowest mean percentage of matriculants (8.5%; 95% CI, 7.2%-9.9%) underrepresented in medicine.

**Conclusions and Relevance:**

In this cross-sectional study, overall US surgical programs had no change in the percentage of applicants or matriculants who self-identified as underrepresented in medicine based on race/ethnicity, but the proportion remained higher than in nonsurgical specialties. Reevaluation of current strategies aimed at increasing racial/ethnic representation appear to be necessary to help close the existing gap in medicine and recruit a more racially/ethnically diverse surgical workforce.

## Key Points

**Question:**

From 2010 to 2018, did the percentage of applicants and matriculants who identified as part of a racial/ethnic group that is underrepresented in medicine change among US surgical specialties?

**Findings:**

In this cross-sectional study evaluating 737 034 applicants and 265 365 matriculants to US residency programs, no statistically significant change was seen in the percentage of applicants or matriculants to most US surgical programs among individuals who identified as part of a racial/ethnic group underrepresented in medicine.

**Meaning:**

The findings suggest that persons belonging to racial/ethnic minority groups continue to be largely underrepresented in surgical fields despite continued efforts to diversify the US surgical workforce.

## Teaser

This cross-sectional study assesses trends in the percentage of applicants and matriculants to US surgical specialties who identified as part of a racial/ethnic group underrepresented in medicine from the 2010-2011 to 2018-2019 academic years

## Introduction

A diverse physician workforce would not only better reflect the increasingly racially/ethnically diverse population of the US but also contribute toward addressing health care disparities, improving patient outcomes and satisfaction, and fostering greater innovation in medicine.^1,2,3^ Despite these observations and numerous initiatives, the percentage of individuals who identify as belonging to racial/ethnic minority groups in medicine has continued to remain below that in the general US population.^4,5^ For example, in 2019, the percentage of the population who identified as Black or African American, American Indian or Alaska Native, Native Hawaiian or other Pacific Islander, and Hispanic or Latino was more than 33%,^6^ while these same groups constituted approximately 12% of US medical school graduates in the class of 2019, without including those who identified as multiracial.^7^

Multiple studies have shown that underrepresentation based on race/ethnicity is also specifically present in surgical fields both among trainees and faculty.^3,8,9,10^ In response, surgical training programs across the US have engaged in initiatives aimed at increasing the number of individuals underrepresented in medicine (URM) based on the definition by the Association of American Medical Colleges (AAMC) as “those racial and ethnic populations that are underrepresented in the medical profession relative to their numbers in the general population.”^11^ However, the impact of these efforts remains unclear.^12,13,14,15^ This study used data from the AAMC to assess trends in the race/ethnicity of applicants and matriculants to surgical specialties between 2010 and 2018.

## Methods

We performed a cross-sectional study using data provided by the AAMC Accreditation Council of Graduate Medical Education residency programs. This study was determined to be exempt from review by the University of Washington institutional review board because it did not involve human participants. Data were deidentified. This study followed the Strengthening the Reporting of Observational Studies in Epidemiology (STROBE) reporting guideline.^16^

Self-reported race/ethnicity (alone or in combination) for applicants and matriculants to surgical and nonsurgical specialties from 2010-2011 to 2018-2019 academic years was obtained and analyzed in April and July 2020. Academic years have been abbreviated to the year that the individual submitted their application to residency (eg, an applicant and matriculant who applied during 2010-2011 is denoted with 2010 rather than the calendar year of matriculation [2011]). Medical specialties were categorized as surgical or nonsurgical based on the definition provided by the American College of Surgeons.^17,18^ Medical specialties considered surgical included general surgery (categorical), neurological surgery, obstetrics and gynecology, orthopedic surgery, otolaryngology, plastic surgery (integrated), thoracic surgery (integrated), urology, and vascular surgery (integrated). Medical specialties considered nonsurgical included anesthesiology, dermatology, emergency medicine, family medicine, internal medicine, neurology, pathology (anatomic and clinical), pediatrics, psychiatry, and radiology (diagnostic). The URM designation followed the AAMC definition and represents individuals who identify as American Indian or Alaska Native; Black or African American; Hispanic, Latino, or of Spanish origin; and Native Hawaiian or other Pacific Islander. Race/ethnicity data were only available for US citizens and permanent residents and include individuals who may have completed their medical education outside the US. White, Asian, other race/ethnicity, unknown race/ethnicity, and non-US citizen or nonpermanent US resident categories were considered non-URM.

### Statistical Analysis

The χ^2^ test for trend was performed to assess changes in proportions across all years between 2010 and 2018 for URM groups and individual races/ethnicities, and the *t* test was performed to evaluate a difference between the mean proportions of individuals who identified as URM in surgical vs nonsurgical specialties using Prism, version 8.4.2 (GraphPad Software). Results were considered statistically significant at a 2-tailed *P* < .05.

## Results

**Figure 1.**
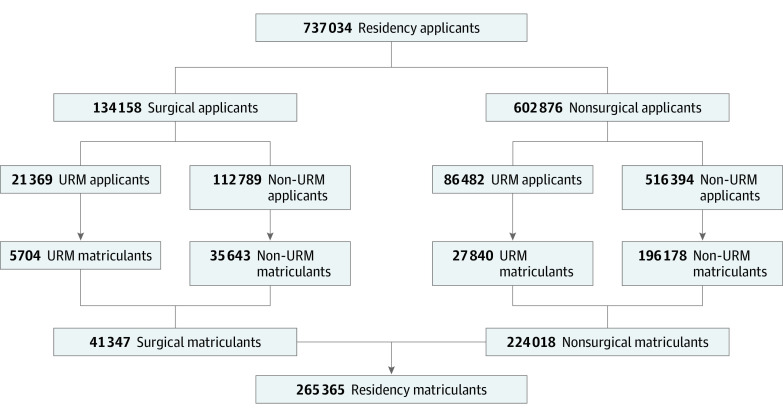
US Residency Applicant and Matriculant Sample by Specialty Type and Underrepresented in Medicine (URM) Status, 2010-2018 Not all residency programs were included in the residency applicant sample; a full list of included residencies is available in the methods. URM refers to individuals who identified as URM based on race/ethnicity as defined by the Association of American Medical Colleges.

A total of 737 034 and 265 365 matriculants during the 9 years of the study period were included in the sample (Figure 1). Of these, 107 851 applicants (14.6%) and 33 544 matriculants (12.6%) identified as URM. A total of 29 724 of 134 158 applicants (22.1%) and 1295 of 41 347 matriculants (3.2%) to US surgical specialties were categorized as other race/ethnicity (self-identified), unknown race/ethnicity, and non-US citizen or nonpermanent US resident. A total of 134 158 applicants (18.2%) applied to surgical specialties, and 602 876 (81.8%) to nonsurgical specialties.

### Trends in Race/Ethnicity Among Applicants

Applicants who identified as URM accounted for 21 369 of the 134 158 surgical applicants (15.9%) from 2010 to 2018. Of the 134 158 surgical residents, 828 (0.6%) identified as American Indian or Alaska Native; 24 481 (18.2%) as Asian; 10 391 (7.7%) as Black or African American; 9955 (7.4%) as Hispanic, Latino, or of Spanish origin; 195 (0.1%) as Native Hawaiian or other Pacific Islander; and 58 584 (43.7%) as White.

**Figure 2.**
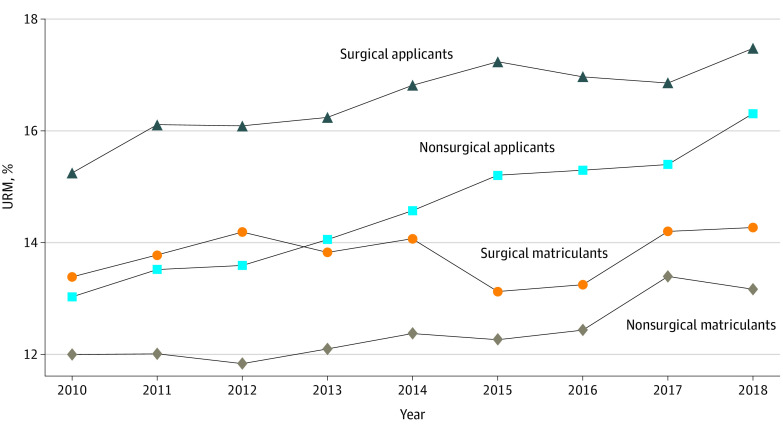
Applicants and Matriculants to US Surgical and Nonsurgical Specialties Who Identified as Underrepresented in Medicine (URM) Based on Race/Ethnicity, 2010-2018

The Table presents the percentage of applicants and matriculants who identified as URM to all surgical specialties combined and to individual surgical specialties in 2010, 2014, and 2018, in addition to the results of the χ^2^ test for trend analyses for the period 2010 to 2018. There was no significant difference in the percentage of applicants who identified as URM in all surgical specialties combined (15.3% [95% CI, 14.7%-15.9%] in 2010 vs 17.5% [95% CI, 16.9%-18.1%] in 2018; *P* = .63). Figure 2 shows trends of applicants and matriculants to surgical specialties combined and nonsurgical specialties combined in the US from 2010 to 2018.

**Table.** Applicants and Matriculants to US Surgical Specialties Who Identified as Underrepresented in Medicine From 2010 to 2018

Specialty	Individuals who identified as underrepresented in medicine				
	Applicants		Matriculants		Representation difference, %
	% (95% CI)	*P* value	% (95% CI)	*P* value	
All surgical specialties					
2010	15.3 (14.7-15.9)	.63	13.4 (12.4-14.4)	.99	−1.9
2014	16.8 (16.2-17.4)		14.1 (13.1-15.1)		−2.7
2018	17.5 (16.9-18.1)		14.3 (13.3-15.3)		−3.2
General surgery					
2010	14.4 (13.4-15.2)	.52	14.0 (12.2-16.0)	.91	−0.4
2014	15.7 (14.9-16.5)		13.7 (11.9-15.6)		−2
2018	16.9 (16.1-17.8)		14.6 (12.8-16.6)		−2.3
Neurological surgery					
2010	11.5 (9.1-14.4)	.88	10.4 (7.0-15.1)	.97	−1.1
2014	12.4 (9.7-15.8)		12.4 (8.8-17.2)		0
2018	13.2 (10.3-16.8)		12.1 (8.4-16.9)		−1.1
Obstetrics and gynecology					
2010	18.5 (17.2-20.0)	.92	18.3 (16.3-20.4)	.87	−0.2
2014	21.5 (20.1-23.0)		20.3 (18.3-22.2)		−1.2
2018	19.4 (18.0-20.9)		17.5 (15.6-19.6)		−1.9
Orthopedic surgery					
2010	13.3 (11.7-15.0)	.58	9.0 (7.2-11.3)	.38	−4.3
2014	13.6 (12.0-15.4)		9.1 (7.3-11.3)		−4.5
2018	14.9 (13.1-16.9)		12.4 (10.2-14.8)		−2.5
Otolaryngology					
2010	11.7 (9.5-11.4)	.40	5.7 (3.6-9.0)	.27	−6.0
2014	12.3 (9.9-15.3)		7.8 (5.3-11.3)		−4.5
2018	16.7 (14.2-19.6)		10.2 (7.5-13.8)		−6.5
Plastic surgery					
2010	12.0 (9.0-16.0)	.28	5.7 (2.2-13.8)	.93	−6.3
2014	15.1 (11.3-19.8)		11.0 (6.7-17.3)		−4.1
2018	17.6 (14.6-21.3)		8.0 (4.9-13.1)		−9.6
Thoracic surgery					
2010	8.1 (4.9-13.2)	.02^a^	0 (0-19.4)	.01^a^	−8.1
2014	15.7 (11.6-21.0)		18.4 (9.2-33.4)		2.7
2018	14.6 (10.2-20.4)		10.0 (4.0-23.1)		−4.6
Urology					
2010	12.0 (9.4-15.3)	.78	12.1 (8.8-16.4)	.80	0.1
2014	13.5 (10.9-16.6)		8.5 (5.9-12.0)		−5.0
2018	12.6 (9.8-15.9)		12.5 (9.4-16.6)		−0.1
Vascular surgery					
2010	13.2 (10.0-17.3)	.20	10.3 (3.6-26.4)	.90	−2.9
2014	14.8 (11.7-18.5)		15.0 (8.1-26.1)		0.2
2018	18.1 (14.6-22.2)		17.2 (9.9-28.2)		−0.9

^a^ Statistically significant based on *P* < .05 using χ^2^ test for trend.

All individual surgical specialties had no significant change in the proportion of applicants who identified as URM from 2010 to 2018 with the exception of thoracic surgery, which nearly doubled from 14 applicants (8.1%; 95% CI, 4.9%-13.2%) in 2010 to 27 applicants (14.6%; 95% CI, 10.2%-20.4%) in 2018 (*P* = .02). There was no significant difference in the percentage of applicants who identified as URM to nonsurgical specialties combined over the study period (13.0% [95% CI, 12.8%-13.3%] in 2010 vs 16.3% (95% CI, 16.0%-16.6%] in 2018; *P* = .43). From 2010 to 2018, obstetrics and gynecology had the highest mean percentage of applicants who identified as URM (20.2%; 95% CI, 19.4%-20.8%) among surgical specialties, and thoracic surgery had the lowest (12.5%; 95% CI, 9.46%-15.4%).

Analysis for each race/ethnicity was consistent with the findings for applicants who identified as URM to surgical specialties combined. No race/ethnicity had a statistically significant change within the applicant pool from 2010 to 2018. Only Hispanic and Latino applicants to thoracic surgery showed a significant increase, from 1.2% (95% CI, 0.2%-4.1%) in 2010 to 5.9% (95% CI, 3.4%-10.3%) in 2018 (*P* = .04). There were no statistically significant differences in the percentage of applicants of each race/ethnicity for nonsurgical specialties combined. From 2010 to 2018, surgical specialties had a significantly higher mean percentage of applicants who identified as URM than did nonsurgical specialties (15.9% [95% CI, 15.4%-16.5%] vs 14.3% [95% CI, 13.5%-15.1%]; *P* = .002).

### Trends in Race/Ethnicity Among Matriculants

**Figure 3.**
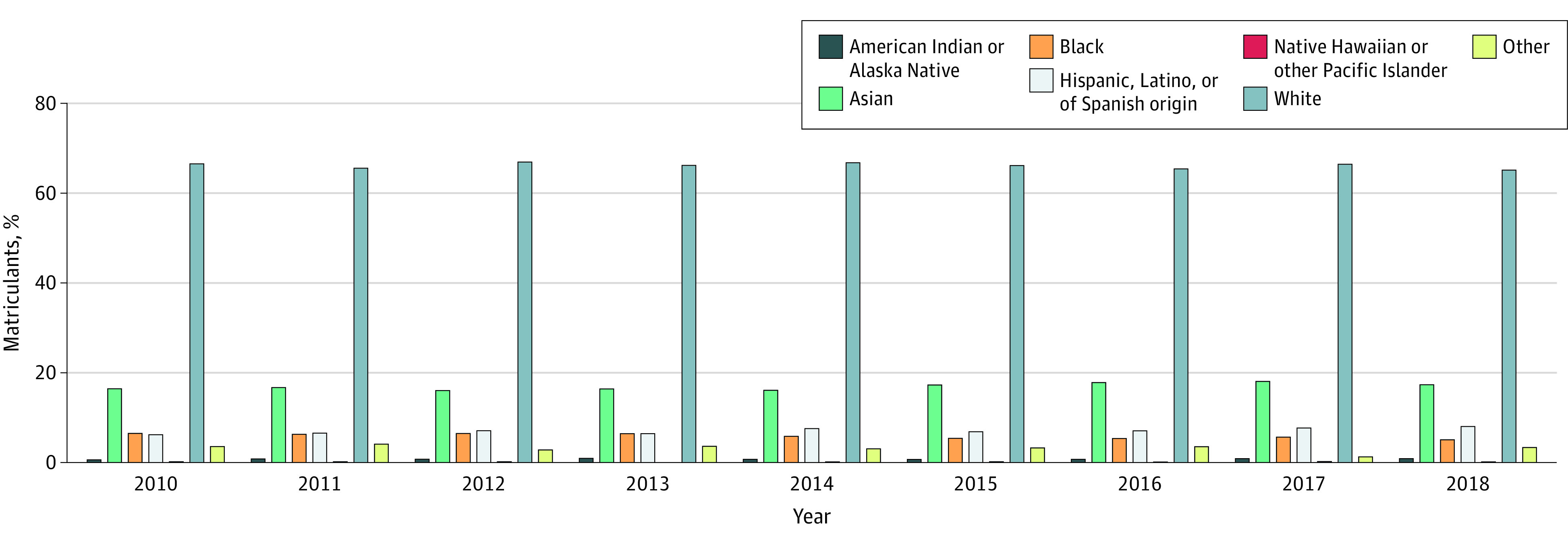
Matriculants to US Surgical Specialties Combined by Race/Ethnicity, 2010-2018 Total percentage may not add to 100% because, starting in 2013 to 2014, individuals were allowed to choose more than 1 racial/ethnic category. Other refers to individuals who chose 1 of the following options: other race/ethnicity, unknown race/ethnicity, non-US citizen, or non–permanent US resident.

A total of 5704 of 41 347 surgical matriculants (13.8%) identified as URM. Compared with the proportion of the applicant pool, Black and African American matriculants (2423 of 41 347 [5.9%]) were the most underrepresented group, and White matriculants (27 352 [66.2%]) were overrepresented by 20.1%. Among the other surgical matriculants, 276 (0.7%) identified as American Indian or Native American; 6996 (16.9%) as Asian; 2925 (7.1%) were Hispanic, Latino, or of Spanish origin; and 80 (0.2%) as Native Hawaiian or other Pacific Islander. Figure 3 shows matriculants to all US surgical specialties combined by race/ethnicity from 2010 to 2018.

The percentage of matriculants who identified as URM did not significantly change for all surgical specialties combined (13.4% [95% CI, 12.4%-14.4%] in 2010 vs 14.3% [95% CI, 13.3%-15.3%] in 2018; *P* = .99). As with applicants, thoracic surgery was the only surgical specialty with an increase in matriculants who identified as URM, with 0% (95% CI, 0%-19.4%) in 2010 and 10.0% (95% CI, 4.0%-23.1%) in 2018 (*P* = .01).

The proportion of surgical matriculants in all racial/ethnic groups did not change significantly from 2010 to 2018 for almost all specialties. The only surgical specialty with a statistically significant change in the percentage of matriculants by race/ethnicity was thoracic surgery. The percentages of Black and African American matriculants increased from 0% (95% CI, 0%-3.7%) in 2010 to 3.0% (95% CI, 0.8%-8.4%) in 2018 (*P* = .03); Hispanic and Latino matriculants from 0% (95% CI, 0%-3.7%) in 2010 to 7.9% (95% CI, 4.1%-14.9%) in 2018 (*P* = .008); Native Hawaiian and other Pacific Islander matriculants from 0% (95% CI, 0%-3.7%) in 2010 to 5.9% (95% CI, 2.8%-12.5%) in 2011 and 0% (95% CI, 0%-3.7%) by 2018 (*P* = .004); and White matriculants from 62.4% (95% CI, 52.6%-71.2%) in 2010 to 65.0% (95% CI, 55.3%-73.6%) in 2018 (*P* = .004). One thoracic surgery matriculant in 2011 self-identified as Native Hawaiian or other Pacific Islander. For matriculants, obstetrics and gynecology had the highest mean percentage of individuals identifying as URM (19.0%; 95% CI, 18.2%-19.8%) among surgical specialties, and otolaryngology had the lowest (8.5%; 95% CI, 7.22%-9.89%).

For nonsurgical specialties, 86 482 of 602 876 applicants (14.3%) and 27 840 of 224 018 matriculants (12.4%) identified as URM (Table). There was no significant difference in percentage of matriculants who identified as URM over the study period for nonsurgical specialties combined (12.0% [95% CI, 11.6%-12.4%] in 2010 vs 13.2% [95% CI, 12.8%-13.6%] in 2018; *P* = .99). Analysis by individual race/ethnicity also did not show a significant change in the percentage of matriculants to all nonsurgical specialties combined. During the study period, surgical specialties had a significantly higher mean percentage of matriculants who identified as URM compared with nonsurgical specialties (13.8% [95% CI, 13.5%-14.1%] vs 12.4% [95% CI, 11.9%-12.6%]; *P* < .001).

### Differences in Representation Among Matriculants Compared With Applicants

In a comparison of the difference between the mean percentages of matriculants and applicants who identified as URM in all surgical specialties combined, there were 2.1% fewer surgical matriculants than applicants (13.8% [95% CI, 13.5%-14.1%] vs 15.9% [95% CI, 15.4%-16.5%]) who identified as URM (Table). In nonsurgical specialties combined, the mean representation difference was −1.9%.

The differences in URM representation increased over time, from −1.3% in 2010 to −2.7% in 2018 for all surgical specialties combined and from −0.8% to −2.8% during the same period for nonsurgical specialties combined. General surgery had a representation difference that increased each year after 2010. The mean difference from 2010 to 2018 between matriculants and applicants who did not identify as URM was 21.1% for surgical specialties (24.3% [95% CI, 19.2%-27.5%] in 2010 and 14.8% [95% CI, 11.7%-17.9%] in 2018) and 17.0% for nonsurgical specialties (21.3% [95% CI, 18.6%-23.9%] in 2010 and 13.6% [95% CI, 10.96%-16.26%] in 2018), with an overall decrease in differences in both surgical and nonsurgical specialties from 2010 to 2018.

## Discussion

Despite efforts to diversify the workforce, our study found no significant change in the percentage of individuals who identified as URM applying or matriculating into the collective 9 American College of Surgeons–recognized surgical specialties from 2010 to 2018. Those who identified as URM accounted for approximately 15.9% of applicants and 13.8% of matriculants to surgical programs in the US from 2010 to 2018. Choinski et al^10^ reported no significant difference in applicants from 2008 to 2018 by race/ethnicity except a significant decrease in Asian applicants to otolaryngology, neurological, vascular, thoracic, orthopedic, and general surgery. The data set used started in 2008, at which time there were more applicants that identified as Asian than in 2010, which may account for the observed trend.

The only surgical specialty with a statistically significant change in our study was thoracic surgery, with a change in both applicants and matriculants, particularly an increase in Hispanic and Latino applicants and in Black and African American, Hispanic and Latino, and Native Hawaiian and other Pacific Islander matriculants. Thoracic surgery had the lowest proportion of applicants (8.1%) and matriculants (0%) who identified as URM in 2010 accompanied with a low total number of individuals who identified as URM; therefore, a small increase in the total number likely indicated a significant increase in the percentage of applicants (14.6%) and matriculants (10.0%) who identified as URM by 2018. Our findings are consistent with previous studies of individual surgical specialties that found no statistically significant increase in the number of individuals who identified as URM in plastic and orthopedic surgery and among otolaryngology applicants.^19,20,21^ Future analysis should consider what happens to applicants who identified as URM who do not matriculate into a surgical program (eg, 2.7% of applicants in 2018) and the implication of these findings.

Although individuals who identify as URM remain underrepresented as applicants and matriculants overall, these proportions are higher than the proportion of those in this group who graduate from medical school (approximately 12% in 2019 excluding those who identified as multiracial).^7^ In all specialties combined, a higher proportion of individuals who identified as URM applied and matriculated into surgical rather than nonsurgical specialties every year between 2010 and 2018 (Figure 2). Prior studies have also found no change in URM representation in nonsurgical specialties, such as radiology (2003-2010), dermatology (1995-2013), and emergency medicine.^22,23,24^ The lack of change suggests that although the US continues to become more diverse, both the surgical and the physician workforces overall have not reflected the racial/ethnic identities of the US population despite increases in available positions, such as the 22% increase in surgical residency positions during the past decade (3470 in 2010 to 4228 in 2018).^25^ This finding differs from the increases observed in surgery among individuals wo identify as URM and women in the 1990s and early 2000s.^26^ Of importance, the stagnant percentage of individuals who identify as URMs in surgery programs suggests that current efforts are falling short in increasing the relative proportions of medical school graduates with varying races/ethnicities and, in particular, those who ultimately decide to pursue surgical programs.

Several national surgical societies have committed to promote diversity through the development of diversity committees and societies for the advancement of specific groups that are URM to increase racial/ethnic representation.^27,28,29,30^ The American College of Surgeons has underscored the importance of moving beyond superficial efforts toward diversity by urging inclusivity, the concept of creating a welcoming space for all health care participants from faculty to patients as individuals and members of the group.^28,31^ Framing recruitment and exposure initiatives in terms of value added over rote diversity metrics will be a necessity to overcoming the inertia of structural bias in creating sustainable growth in the proportion of surgical subspecialty training matriculants from URM backgrounds.^32^

The difference in representation of matriculants compared with applicants who identify as URM in surgical specialties increased from 2010 to 2018 (Table). Individuals who did not identify as URM were also overrepresented by a mean of 20.1% compared with their proportion of the applicant pool. This was likely attributable to the 22.0% mean difference in representation between White matriculants and applicants because the differences for Asian, other race/ethnicity, unknown race/ethnicity, and non-US citizen or non–permanent US resident were less than 1.0% and often negative for each cycle. Orthopedic, plastic, and vascular surgery were the only specialties in which there was a smaller difference in URM representation for most of the 8 years after 2010 owing to an increase in matriculation of individuals who identified as URM; however, these specialties had more applicants who identified as URM than matriculants in this group, as has been reported previously for plastic surgery.^19^ The difference in representation affects the entire training pathway: 14.9% of medical school applicants, 13.6% of medical school matriculants, 12.4% of 2019 graduates, 17.0% of surgical residency applicants, 14.3% of surgery residency matriculants, and 12.8% of practicing surgeons in the US identified as URM during the 2018-2019 academic year.^7,33,34,35,36^

Although large disparities in gender among surgical trainees remain, the strategies used to increase representation from 15% in 2000 to 25% by 2013 may provide some insight for improving racial/ethnic diversity in the surgical workforce.^37,38^ Diversity within a program has been shown to play some role in program selection by applicants, among multiple other factors, as has been shown in studies evaluating residency program choices by women and individuals who identify as URM.^39,40^ Some strategies that have been successful in increasing the representation of women and, to a certain extent, groups who identify as URM include meaningful mentorship experiences, changes in the culture of surgical departments, and shifts in the focus of residency interviews.^41^

Directed exposure and longitudinal relationship-building efforts, such as the Diverse Surgeon’s Initiative and The Harold Amos Medical Faculty Development Program through the Robert Wood Johnson Foundation have been shown to be successful frameworks for providing skills and resources for applicants and trainees who identify as URM to advance in all stages of career development in academic surgical fields.^15,42^ Early exposure to surgical specialties through strategic support initiatives, such as summer internship programs, may help to describe these fields and establish mentorship relationships, which have been associated with significant increases in applicants who identify as URM.^13,14^ Cochran et al^43^ described a grounded theory model of successful mentorship in academic surgery that emphasizes the need for multiple mentors to engage with a diverse group of mentees over time, covering unique strategic domains as opposed to overreliance on fixed mentorship dyads.

There are also steps during the application process that should be studied further and potentially restructured. Websites are an underused way of communicating a program’s commitment to diversity and could be enhanced to further increase diversity among applicants.^44^ Receiving an interview offer has been recognized as an important step of gatekeeping the number of applicants who identified as URM.^20^ A study assessing general surgery programs found that having more residents and faculty who identify as URM was not associated with an increased likelihood of applicants who identified as URM being offered an interview.^45^ Creative and new strategies should be developed to encourage more applicants, make the interview process more equitable, and decrease the overall attrition of individuals who identify as URM who matriculate.^12,46,47,48^ Continued active evaluation in a reiterative way will be needed to identify strategies that are or are not helping to diversify the workforce.

Addressing the work environment experienced by residents who identify as URM may improve the resident experience as well as influence the encounters of medical students who identify as URM during their clinical rotations and their perception of surgery.^49,50^ Studies about women identified that the environment of training affects why students choose to pursue surgery.^51,52^ Recent reports detail the discrimination and harassment that individuals who identify as URM and women experience in surgical training, which has been disavowed by the American Surgical Assocation.^53,54^ Further change in hierarchical and social dynamics is likely necessary to increase the number of individuals who identify as URM who ultimately pursue a surgical field.

### Limitations

This study has limitations. We used self-report data, which relies on participants’ willingness to accurately share personal information. Race is a complex social category with evolving meaning, shown by how AAMC permitted students to select more than 1 race/ethnicity after 2013. This change means that data after 2013 with race/ethnicity alone and in combination counts multiracial individuals in more than 1 category, potentially overestimating the percentage of students who identify as URM. We did not have access to identify which applicants who identified as URM attended osteopathic or non-US medical schools, which may be a confounder because these students tend to have lower matriculation rates into US residency programs, including surgical ones.^55,56^ Because our focus was on surgical specialties, we did not investigate the trends for individual nonsurgical specialties; perhaps there is insight that can be gained from that analysis in future studies.

## Conclusions

In this cross-sectional study, there was no change in the proportion of individuals who identified as URM applying and matriculating to surgical and nonsurgical specialties overall from 2010 to 2018. These findings support the claim that underrepresentation of racial/ethnic minorities is a broader issue that extends beyond surgery, starting from entry into medical school and beyond residency. Therefore, to reach the goal of accurately representing the demographics of the US and improving patient care in surgery, further investment in innovative programs focused on increasing racial/ethnic diversity from the medical school to the surgical residency level appears to be needed.
